# Editorial: Targeting the Wnt/β-catenin signaling pathway in cancer, volume II

**DOI:** 10.3389/fonc.2026.1970244

**Published:** 2026-09-03

**Authors:** Simone Patergnani, Donald J. Buchsbaum, Gary A. Piazza, Gang Zhou

**Affiliations:** 1 Department of Medical Sciences, Laboratory for Technologies of Advanced Therapies, University of Ferrara, Ferrara, Italy; 2 Department of Obstetrics and Gynecology, University of Alabama at Birmingham, Birmingham, AL, United States; 3 Department of Drug Discovery and Development, Harrison College School of Pharmacy, Auburn University, Auburn, AL, United States; 4 Georgia Cancer Center, Augusta University, Augusta, GA, United States

**Keywords:** cancer therapeutics, molecular targets, signaling, targeted therapy, Wnt/β-catenin

## Introduction

Canonical Wnt/β-catenin signaling lies at the crossroads of embryonic development, tissue homeostasis, and cancer. Under physiological conditions, this pathway regulates essential processes such as proliferation, cell fate, migration, survival, and stem cell renewal. In cancer, constitutive activation of Wnt/β-catenin signaling, for example, from mutations or aberrant overexpression of pathway components, can promote malignant transformation and sustain tumor growth, stem-like features, and interactions with the surrounding microenvironment. Wnt/β-catenin signaling can also influence tumor-immune interactions, as demonstrated by its association with dendritic cell dysfunction and T-cell exclusion in cancers.

This dual role in maintaining cellular homeostasis, while being aberrantly activated in cancer has long complicated the therapeutic targeting of Wnt/β-catenin signaling. The challenge is not simply to inhibit the pathway, but how to selectively interfere with its oncogenic functions while preserving its essential roles in normal tissues to mitigate on-target toxicities.

**Figure 1:**
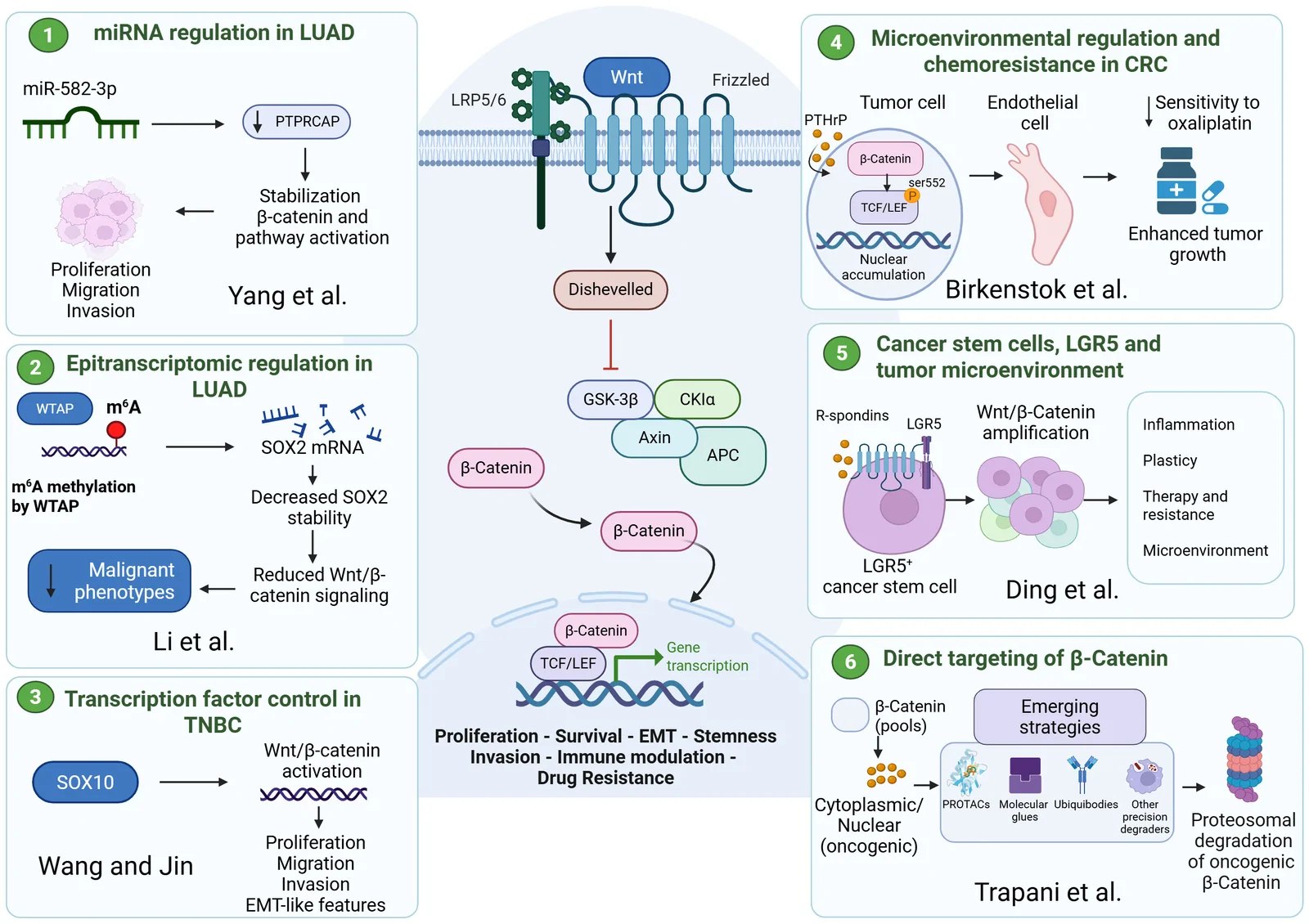
Multilevel regulation and therapeutic targeting of Wnt/β-catenin signaling in cancer. Schematic overview of the molecular and cellular mechanisms highlighted across the six contributions to this Research Topic. In lung adenocarcinoma (LUAD), miR-582-3p promotes Wnt/β-catenin pathway activation through suppression of PTPRCAP, whereas WTAP-mediated m6A modification decreases SOX2 mRNA stability and limits SOX2-dependent Wnt/β-catenin signaling. In triple-negative breast cancer (TNBC), SOX10 acts as an upstream transcriptional regulator associated with Wnt/β-catenin activation and malignant phenotypes. In colorectal cancer (CRC), PTHrP promotes Ser552 phosphorylation and nuclear accumulation of β-catenin and is associated with reduced oxaliplatin sensitivity, including effects mediated by the tumor microenvironment. LGR5 represents a Wnt-responsive cancer stem-cell marker and R-spondin receptor that links pathway amplification with stemness, inflammation, tumor microenvironment interactions, and therapeutic resistance. Finally, emerging β-catenin-directed approaches, including PROTACs and other precision degraders, aim to preferentially eliminate oncogenic cytoplasmic and nuclear β-catenin while preserving the cadherin-associated membrane pool required for cell-cell adhesion. Together, these studies emphasize that Wnt/β-catenin signaling is controlled at multiple regulatory levels and cellular compartments, supporting context-specific and biomarker-guided therapeutic strategies. APC, adenomatous polyposis coli; CK1, casein kinase 1; CRC, colorectal cancer; EMT, epithelial-to-mesenchymal transition; GSK3β, glycogen synthase kinase 3 beta; LGR5, leucine-rich repeat-containing G protein-coupled receptor 5; LRP5/6, low-density lipoprotein receptor-related protein 5/6; LUAD, lung adenocarcinoma; m6A, N6-methyladenosine; miRNA, microRNA; PROTAC, proteolysis-targeting chimera; PTHrP, parathyroid hormone-related peptide; PTPRCAP, protein tyrosine phosphatase receptor type C-associated protein; SOX2, SRY-box transcription factor 2; SOX10, SRY-box transcription factor 10; TCF/LEF, T-cell factor/lymphoid enhancer factor; TNBC, triple-negative breast cancer; WTAP, Wilms’ tumor 1-associating protein. Created in https://BioRender.com.

The six contributions included in this Research Topic illustrate this complexity from different perspectives. Together, they show that Wnt/β-catenin activity can be shaped by non-coding RNAs, epitranscriptomic regulation, transcription factors, signals from the tumor microenvironment, cancer stem-cell states, and direct regulation of β-catenin itself. Rather than pointing to a single therapeutic solution, these studies suggest that the most effective strategy may depend on how, where, and at what stage of disease the pathway becomes dysregulated (**Figure 1**).

Two studies explore upstream regulatory mechanisms in lung adenocarcinoma (LUAD). Yang et al. identified PTPRCAP as a direct target of miR-582-3p. By suppressing PTPRCAP, miR-582-3p promotes Wnt/β-catenin activation, thereby enhancing proliferation, migration, and invasion in LUAD cells. Conversely, restoration of PTPRCAP counteracts these effects. These findings connect post-transcriptional regulation to Wnt pathway activity and suggest that both miR-582-3p and PTPRCAP may have value as molecular markers or therapeutic targets.

Li et al. describe an additional layer of post-transcriptional regulation in LUAD. They show that Wilms’ tumor 1-associating protein (WTAP) promotes N6-methyladenosine (m6A) modification of SRY-box transcription factor 2 (SOX2) mRNA, reducing its stability and consequently lowering SOX2 expression. This attenuates Wnt/β-catenin signaling and suppresses malignant phenotypes in LUAD cells. These findings identify epitranscriptomic regulation as an additional mechanism controlling Wnt/β-catenin signaling, with potential implications for biomarkers and therapy.

Wang and Jin show that SOX10 promotes proliferation, migration, invasion, and epithelial-to-mesenchymal transition (EMT)-like changes in triple-negative breast cancer (TNBC) cells by activating Wnt/β-catenin signaling. Pharmacological activation or inhibition of the pathway modulates the effects of SOX10, supporting Wnt/β-catenin signaling as a key mediator of SOX10-driven tumor aggressiveness. These findings illustrate how transcription factors may shape Wnt dependence in specific tumor contexts, although further validation in animal models and patient samples will be important.

Birkenstok et al. extend the analysis of Wnt/β-catenin signaling to the tumor microenvironment and treatment response in colorectal cancer (CRC). They show that parathyroid hormone-related peptide (PTHrP) promotes Ser552 phosphorylation and nuclear accumulation of β-catenin and is associated with reduced sensitivity to oxaliplatin. This effect is observed both directly in tumor cells and indirectly through signals derived from endothelial cells. Interestingly, the response to oxaliplatin differs between xenograft and syngeneic models, indicating that the impact of PTHrP on chemoresistance is context-dependent and may be influenced by the surrounding tumor microenvironment.

Ding et al. provide a broader view of the field through a bibliometric analysis of research on leucine-rich repeat-containing G protein-coupled receptor 5 (LGR5), a well-established Wnt target and marker of stem-cell populations. LGR5 also functions as a receptor for R-spondins, thereby enhancing Wnt/β-catenin signaling. Their analysis identifies LGR5-positive cancer stem cells, inflammation, and the tumor microenvironment as emerging areas of interest, highlighting the growing attention to the interplay between Wnt signaling, cancer stemness, and the surrounding tumor niche.

Finally, Trapani et al. focus on the therapeutic challenge of directly targeting β-catenin. β-catenin remains a difficult pharmacological target because it lacks conventional drug-binding pockets and also plays an essential structural role in cadherin-mediated cell adhesion. The authors discuss emerging strategies, including proteolysis-targeting chimeras (PROTACs), molecular glues, ubiquibodies, and other precision degraders. Of particular interest is the possibility of selectively degrading the oncogenic cytoplasmic and nuclear pools of β-catenin while preserving the membrane-associated pool required for adherens junctions. This approach may help overcome one of the major limitations of β-catenin-directed therapies by improving tumor selectivity while reducing on-target toxicity.

Taken together, the articles in this Research Topic reinforce the idea that Wnt/β-catenin signaling should not be viewed as an isolated linear pathway. Its activity is shaped by multiple regulatory mechanisms and by the cellular and microenvironmental context in which tumor cells reside. Understanding these different levels of regulation will be essential for developing more selective and effective therapeutic strategies.

## Perspectives

The future of Wnt/β-catenin-targeted therapy will likely rely on precision rather than broad pathway inhibition. Identifying the mechanisms, cellular populations, and β-catenin pools that specifically sustain tumor growth will be crucial to preserve physiological signaling while maximizing antitumor activity. Biomarker-guided and context-specific strategies may therefore be key to achieving safer and more effective therapeutic targeting.

